# Case Report: Minimally invasive management of torsion in a giant ovarian cyst in an obese child: a single-port approach combined with extracorporeal cystectomy

**DOI:** 10.3389/fmed.2026.1731230

**Published:** 2026-03-16

**Authors:** Ying Yang, Yintong Wang, Jing Sun, Chuman Zhang, Yue Yang

**Affiliations:** 1 Dalian Medical University, Dalian, China; 2 Department of Gynecology, Dandong Central Hospital, Dalian, China

**Keywords:** adolescent gynecology, case report, extracorporeal ovarian cystectomy, fertility-sparing surgery, ovarian torsion, pediatric gynecology, single-port laparoscopy

## Abstract

**Background:**

Ovarian torsion in children is most commonly associated with benign cystic lesions. The surgical objective is to achieve early detorsion while maximizing ovarian function preservation.

**Case Presentation:**

We report a case of an 11-year-and-8-month-old obese girl who had not yet reached menarche. She presented with persistent right lower abdominal pain lasting 40 h. Ultrasound revealed a large right-sided cystic mass with a “whirlpool sign” and an O-RADS 2 classification. CT imaging demonstrated a sizable pelvic cystic lesion with minimal fluid accumulation. Upon admission, ALT and AST levels were 278 and 122 U/L, respectively. Following a multidisciplinary evaluation, a perioperative management plan was devised, focusing on minimizing hepatic metabolic burden. Single-port laparoscopic surgery via the umbilicus confirmed 540° torsion of the right adnexal pedicle. After detorsion, an external ovarian cystectomy and ovarian reconstruction were performed outside the protective sleeve, involving controlled decompression, complete cyst wall excision, meticulous hemostasis, and tissue reconstruction. Intraoperative blood loss was approximately 20 ml. Postoperatively, transaminase levels gradually normalized, and the patient was discharged on postoperative Day 4. Histopathology revealed an ovarian follicular cyst with mild granulosa cell hyperplasia.

**Conclusions:**

Combining single-port laparoscopy with extracorporeal cystectomy provides a safe, minimally invasive solution for pediatric ovarian torsion. This approach reduces operative time, minimizes CO_2_ exposure, and preserves both ovarian function and appearance, making it a reproducible option for pediatric cases.

## Introduction

Ovarian follicular cysts are common gynecological conditions that typically result from the failure of immature follicles to develop or from anovulation, leading to unruptured or atretic follicles that continue to enlarge. When a follicle persists and exceeds 3 cm in diameter, it is classified as an ovarian follicular cyst. Solitary follicular cysts can occur at various stages of a woman's life, particularly during childhood, adolescence, and the reproductive years (1). Their diameters generally range from 3 cm to 8 cm, with cysts exceeding 8 cm being relatively uncommon. Because symptoms are often subtle or nonspecific, larger follicular cysts may undergo torsion due to sudden changes in body position, resulting in acute abdominal pain. Delayed diagnosis and treatment can have serious consequences for the patient's health. Adolescent girls are particularly vulnerable to this condition. Studies have shown that approximately 52% of ovarian torsion cases in children occur between the ages of 9 and 14 years, with a median age of 11 years (2, 3). This report presents a case involving an 11-year-old obese girl who was postoperatively diagnosed with torsion of a giant ovarian follicular cyst.

## Case presentation

### Case description

The patient was an 11-year-and-8-month-old unmarried, nulliparous girl with no menarche. She was admitted to the emergency department at 15:00 on September 3, 2024, presenting with a 6-month history of a pelvic mass and 40 h of right lower abdominal pain. Six months earlier, ultrasonography at this hospital had revealed a cystic mass in the right adnexal region measuring approximately 6.7 × 6.0 × 3.6 cm. A follow-up examination five months prior showed the mass to be approximately 4.8 × 3.9 × 3.2 cm, and observation was recommended. The initial abdominal pain resolved spontaneously but recurred the following morning after breakfast, manifesting as paroxysmal pain. A CT scan performed at another hospital 3 h before admission revealed multiple cystic lesions in the pelvis (largest approximately 9.4 × 7.9 cm) with minimal fluid accumulation, suggestive of an ovarian tumor with possible torsion. The patient was subsequently transferred to our hospital. Physical examination: height 168 cm, weight 85 kg, BMI 30.12 kg/m^2^.

Admission findings: temperature 36.3 °C, pulse 91 bpm, respiratory rate 18 bpm, blood pressure 130/78 mmHg. The abdomen was soft and flat, with tenderness in the right lower quadrant but no rebound tenderness or muscle guarding. Combined recto-abdominal palpation identified an approximately 8 cm mass in the right adnexal region with well-defined borders, limited mobility, and tenderness. The uterus was slightly small and freely mobile, and no significant abnormalities were noted in the left adnexa.

**Figure 1:**
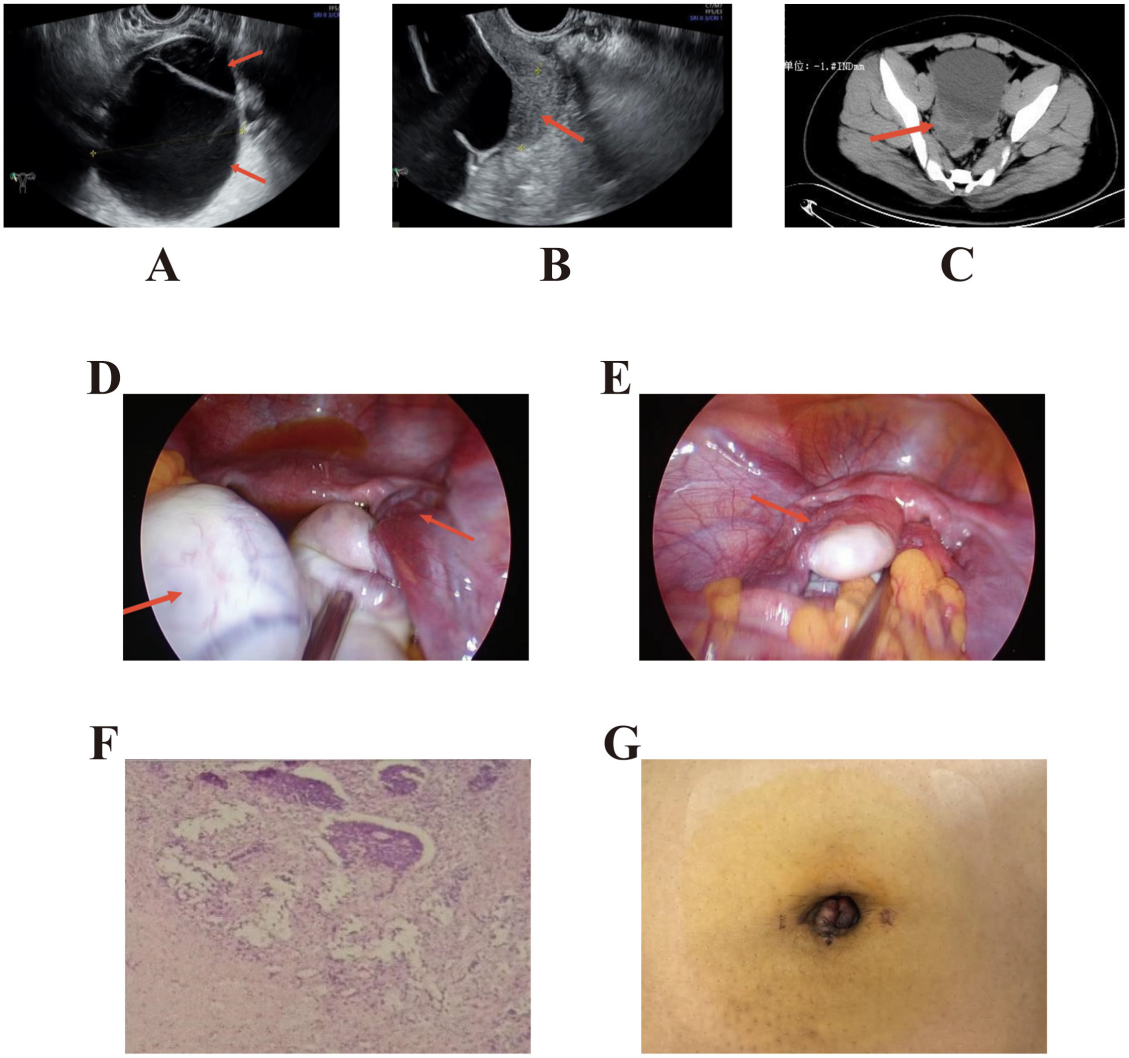
**(A)** Ultrasound on admission day showing a large cystic mass in the right adnexal region, approximately 12.0 × 9.4 × 9.1 cm in size, with clear margins, anechoic interior, and visible septal reflections. **(B)** Dynamic ultrasound observation revealing a “whirlpool sign.” A mixed-echo mass, measuring approximately 4.8 × 4.7 cm, was observed between the cyst and the uterine body. Real-time imaging demonstrated whirl-like changes, indicating pedicle torsion. **(C)** Whole-abdominal CT from another hospital showing a pelvic cystic mass with pelvic effusion and fatty liver changes. **(D)** Single-port laparoscopic exploration revealing partial cystic enlargement of the right ovary (approximately 12 × 10 cm), with an additional 2 × 1 cm protruding cyst. Right adnexal torsion was observed at 540°, with minimal pelvic fluid. **(E)** Intraoperative view showing the right ovary reduced into the pelvis following extracorporeal manipulation, complete cyst wall dissection, and suture reconstruction after ovarian plication. **(F)** Postoperative pathology showing a right ovarian follicular cyst with mild granulosa cell hyperplasia. **(G)** Postoperative umbilical incision appearance after cosmetic suturing using the “anchor fixation technique”.

Auxiliary investigations: ultrasonography revealed a large cystic mass in the right adnexal region measuring approximately 12.0 × 9.4 × 9.1 cm (Figure 1A). A 4.8 × 4.7 cm mixed-echo mass was observed between the uterus and the cystic lesion, showing a “whirlpool sign” on dynamic imaging (Figure 1B). A 1.8 cm fluid-filled hypoechoic area was noted in the rectouterine pouch. ECG: Sinus rhythm, ST-T changes, and left ventricular hypertrophy. Laboratory tests: ALT 278 U/L, AST 122 U/L, blood glucose 8.9 mmol/L, total cholesterol 5.66 mmol/L, triglycerides 3.93 mmol/L. because the patient was transferred from a referring hospital, tumor markers had been obtained prior to transfer, and the results were reviewed upon arrival in our emergency department. Tumor markers were within normal ranges (CA-125 10.90 U/ml, CA19–9 5.96 U/mL, AFP 1.81 ng/ml, CEA 1.23 ng/ml); coagulation function normal; AMH 6.36 ng/ml. Serum β-hCG was not measured in this prepubertal patient. Abdominal CT (Figure 1C) from the referring hospital showed a pelvic cystic mass, pelvic effusion, and fatty liver. Admission diagnosis: Pelvic mass (suspected ovarian tumor with torsion).

Perioperative management: based on an ultrasound O-RADS 2 classification and strong suspicion of ovarian torsion, emergency laparoscopic exploration was scheduled. Due to markedly elevated transaminase levels, a gastroenterology consultation diagnosed hepatic impairment and recommended discontinuation of previous medications, avoidance of hepatotoxic drugs, and initiation of hepatoprotective therapy. The anesthesiology team planned spinal-epidural combined anesthesia with short-duration general anesthesia to minimize hepatic metabolic load. Single-port laparoscopic surgery was performed the same day under intravenous sedation combined with spinal-epidural anesthesia.

### Intraoperative findings and surgical procedure

A single-port umbilical approach was employed using a multi-channel single-port device. Upon laparoscopic entry, a systematic exploration of the abdominal cavity was first performed, and the appendix was visualized without gross inflammatory changes suggestive of appendicitis (e.g., erythema, edema, exudate, or perforation). Intraoperative findings included a slightly small uterus with a smooth surface and a partially cystically enlarged right ovary measuring approximately 12 × 10 cm, with an additional 2 × 1 cm protruding cyst. The right adnexal pedicle was twisted 540° (Figure 1D), with minimal pelvic fluid observed. After detorsion, the right ovary was confirmed to have viable tissue. The ovary was then elevated to the umbilical incision for controlled puncture decompression. An external ovarian cystectomy was performed using the PORT protective cover. The cyst wall was completely excised and submitted for frozen section analysis, which indicated a benign lesion. The cyst base was closed with interrupted 3–0 absorbable sutures, followed by continuous suturing to restore the ovarian contour. The smaller cyst was excised at its pedicle, and the defect was sutured. After meticulous hemostasis at all operative sites, the ovary was repositioned, the pelvic cavity was irrigated and suctioned, and residual CO_2_ was evacuated (Figure 1E). Finally, an “anchor technique” was used for cosmetic umbilical closure. The operation proceeded uneventfully, the estimated blood loss was approximately 20 ml, mainly attributable to minor bleeding from the umbilical incision/access site and slight oozing from the ovarian hilum/parenchyma during cyst enucleation and ovarian reconstruction; hemostasis was achieved routinely without transfusion, intraoperative fluid replacement of 1,000 mL, no transfusion required, and a urine output of 100 ml.

### Postoperative course and outcome

Postoperative recovery was uneventful, and symptoms were well controlled. On postoperative Day 1, follow-up biochemical testing showed ALT and AST levels decreased to 214 U/L and 88 U/L, respectively. The patient passed flatus approximately 30 h after surgery. On Day 2, the urinary catheter was removed, and a semi-liquid diet was initiated. Bowel function returned on Day 3, with further reductions in ALT and AST levels to 136 U/L and 53 U/L, respectively. By Day 4, the incision demonstrated Grade I/A healing, and the patient was discharged. Final histopathological examination confirmed a right ovarian follicular cyst with mild granulosa cell hyperplasia (Figure 1F). The total hospitalization period was four days, with no postoperative complications, continuous improvement in liver enzyme levels, benign pathology, and a satisfactory cosmetic outcome. Discharge diagnoses: (1) Torsion of a right ovarian follicular cyst; (2) Hepatic impairment.

### Follow-up

At approximately 12 months postoperatively, follow-up evaluation showed no recurrence of torsion and no need for reoperation. The patient had not yet experienced menarche. Pelvic ultrasonography revealed a right ovary measuring approximately 4.0 × 3.0 × 2.0 cm, with an estimated volume of 12 ml based on the ellipsoid formula. The ovarian cortex appeared well preserved, with normal blood flow signals observed. No residual cavities or recurrent cystic lesions were detected. The contralateral ovary demonstrated normal morphology and vascularity. The umbilical incision scar appeared satisfactory, with no evidence of infection, incisional hernia, or other postoperative complications.

## Discussion

Single-incision (single-port) transumbilical laparoscopic-assisted extracorporeal ovarian cystectomy has been previously reported in pediatric patients (4, 5). Ovarian follicular cysts lack distinctive clinical features compared with other ovarian tumors and often present without characteristic symptoms or signs. Menstrual irregularities are the most common manifestation. Some patients may experience abdominal pain, abdominal distension, or a palpable mass, which can easily lead to missed or incorrect diagnoses. In pediatric patients, symptoms of precocious puberty such as vaginal bleeding or breast development may occur. When cysts enlarge and lead to complications such as torsion or rupture, they can cause acute abdominal pain, prompting medical evaluation (6). In the present case, the patient's primary symptom was right lower quadrant pain following a change in body position. Ultrasonography revealed a mixed-echo mass between the adnexa and the uterine body, displaying a “whirlpool sign” during dynamic imaging—an established and reliable sonographic indicator of ovarian torsion (7, 8). Therefore, emergency surgical intervention was undertaken.

### Preoperative preparation

The patient, an 11-year-and-8-month-old girl with a BMI greater than 30 kg/m^2^, required careful consideration in selecting an appropriate emergency surgical approach. Several key factors were taken into account:

①The patient's young age indicated incomplete physical maturation, which limited intra-abdominal operating space and surgical maneuverability. The increased subcutaneous fat layer associated with obesity further complicated the procedure.

②At the time of surgery, the patient had been experiencing abdominal pain for more than 36 h, necessitating intraoperative evaluation to determine whether ovarian preservation was feasible.

③The exact location and nature of the lesion could only be confirmed during intraoperative exploration, which would guide the choice of specific surgical technique.

④The mass was large and cystic in nature. To ensure surgical safety, improve postoperative recovery, and minimize physical and psychological distress, laparoscopic exploration was planned (9, 10). Preoperatively, a single-port laparoscopic approach via the umbilicus was selected to avoid the complex intra-abdominal maneuvers associated with conventional multi-port laparoscopy (11, 12).

### Intraoperative management of ovarian tumor torsion

Following the restoration of normal anatomical structures during surgery, both fallopian tubes appeared normal, and the right ovary exhibited viable ovarian tissue. A right ovarian tumor enucleation was subsequently performed. Traditionally, the management of ovarian torsion has involved excision of the affected adnexa. In such cases, the tumor and twisted pedicle are resected after clamping below the pedicle base, and detorsion is avoided before clamping to prevent potential embolus dislodgement and pulmonary embolism. However, recent studies have demonstrated that performing tumor enucleation after detorsion is equally safe and effective, with no significant difference in the incidence of pulmonary embolism compared to the conventional approach (13). Furthermore, the detorsed ovary often retains normal function during long-term follow-up, even when its appearance is abnormal. Some cases of ovarian torsion present with venous congestion; however, due to the ovary's dual blood supply, the risk of necrosis remains low. The characteristic blue-black discoloration commonly observed is typically attributable to venous stasis rather than irreversible ischemia. Malignant ovarian tumors are exceedingly rare in children, and the likelihood of malignancy associated with torsion is even lower (14–16). Therefore, oophorectomy should not be routinely performed for enlarged, edematous, or twisted ovaries. In summary, the decision to remove the ovary intraoperatively should be guided by the nature of the cyst. Ovarian preservation or excision should not be based solely on the gross appearance of the twisted adnexa but should instead consider multiple factors, including symptom duration, the presence of preoperative infection, intraoperative adhesions, and evidence of malignancy such as peritoneal nodules (17).

### Excision of the umbilical stump and suturing

Intraoperative observation revealed that the patient's ovarian ligaments were relatively long (18). Given her slow growth rate and short stature, the ovary was gently elevated outside the umbilical ring to facilitate tumor enucleation and suturing. This technique shortened operative time, reduced collateral tissue damage caused by excessive traction, and decreased the overall surgical difficulty, making it particularly suitable for pediatric patients. Moreover, this approach adheres to the principles of tumor-free surgery, allowing timely lesion identification, effective hemostasis, and maximal preservation of functional ovarian tissue (19). During extracorporeal suturing, maintaining a non-CO_2_ pneumoperitoneum helps minimize systemic CO_2_ absorption. Previous studies have shown that obese patients are prone to airway hypertension and reduced lung compliance (20), with intraoperative PETCO_2_ and PCO_2_ levels tending to increase and pH levels to decrease. Despite the enhanced compensatory capacity observed in obese individuals, intraoperative procedures should be completed as efficiently as possible to prevent hypercapnia resulting from CO_2_ accumulation (21). This approach also minimizes anesthetic use and reduces the adverse effects of prolonged anesthesia on pediatric patients.

### Management of abnormal liver function

Routine admission testing revealed elevated transaminase levels. The patient had a history of medication use during previous treatment for an unspecified condition. Following consultation with the Gastroenterology Department, liver injury was diagnosed, although the underlying cause remained uncertain. The following potential etiologies could not be excluded: (1) fatty liver–related, (2) drug-induced, (3) secondary to acute disease–related stress, or (4) other causes. An anesthesiology consultation was obtained preoperatively. During surgery, the anesthesiologist administered combined intravenous and spinal-epidural anesthesia, carefully selecting anesthetic agents to minimize hepatic metabolic load (22). This approach optimized surgical safety while minimizing perioperative risk. Postoperatively, liver function should be monitored closely to detect abrupt deterioration and to mitigate potential complications. In cases involving emergency management of unexplained hepatic dysfunction in pediatric patients, multidisciplinary consultation is essential to achieve a comprehensive understanding of the patient's condition and to formulate an appropriate perioperative management plan. Emergency surgical intervention in the setting of abnormal liver function can be safely performed when properly managed. The combination of laparoscopic minimally invasive techniques and enhanced recovery after surgery protocols can effectively attenuate the stress response and mitigate postoperative declines in serum albumin. Intraoperative warming and postoperative fluid restriction have been shown to reduce postoperative albumin loss and alleviate hepatic stress (23).

### Navel “cosmetic procedures”

For umbilical repair, the “anchor technique” was employed for cosmetic closure of the umbilicus (Figure 1G), strictly adhering to sterile and minimally invasive surgical principles. This method effectively reduces the risk of complications such as umbilical hernia and delayed wound healing, while offering short operative duration, minimal scarring, and favorable umbilical contouring outcomes (24). Regarding postoperative pain management, local infiltration anesthesia administered prior to umbilical puncture can partially alleviate postoperative discomfort and facilitate recovery. This aspect represents an area for further refinement in clinical practice.

In summary, the management strategy for this case centered on “early detorsion combined with a single-port approach and extracorporeal cystectomy with ovarian reconstruction,” which demonstrated distinct advantages in the emergency management of pediatric ovarian torsion. These included shortening intra-abdominal manipulation and pneumoperitoneum time, minimizing traction and instrument interference, facilitating meticulous hemostasis and suturing, maximizing ovarian cortex preservation, and achieving satisfactory cosmetic results. Moreover, individualized perioperative management was integrated to balance surgical efficiency and safety.

It is important to note that this report describes a single case with limited follow-up, and its generalizability requires further validation. This technique is best suited for lesions identified on imaging as low-risk, cystic, and well-defined, which introduces a certain degree of selection bias. Extracorporeal cystectomy and ovarian reconstruction require substantial technical expertise, involving a learning curve and a potential risk of cyst fluid leakage. The necessity of ovarian fixation remains a subject of ongoing debate.

In obese pediatric patients, single-port laparoscopy may be technically challenging due to limited working space and concerns regarding mobilization and extracorporeal cystectomy. In this case, the umbilicus (typically the thinnest portion of the abdominal wall) allowed safe access via an open umbilical incision, and the adnexa could be gently elevated to the umbilical ring after controlled cyst decompression; moreover, the relatively short umbilicus–pubis distance in children facilitated extracorporeal enucleation and reconstruction. If exteriorization is not feasible because of limited reach or excessive tension, cystectomy and ovarian reconstruction can be completed intracorporeally using established single-port techniques without necessarily increasing procedural complexity. Therefore, with appropriate patient selection and contingency planning, a single-port strategy can be a feasible and adaptable option even in obese pediatric patients.

## Conclusion

This case highlights the presentation of acute abdominal pain in children complicated by adnexal cystic lesions. Upon detection of the “whirlpool sign,” early laparoscopic exploration should be performed, with ovarian preservation as the primary objective. For large cysts with imaging findings suggestive of benignity that require morphological reconstruction, a single-port approach combined with extracorporeal cystectomy offers a balanced solution, optimizing surgical efficiency, preserving ovarian tissue, and achieving favorable cosmetic outcomes.

## Patient perspective

Her guardians were mainly worried that the acute abdominal pain indicated ovarian torsion and that surgery might compromise her future fertility. They were also concerned about anesthesia safety given elevated liver enzymes, possible heavy bleeding, and postoperative scarring.

## Data Availability

The original contributions presented in the study are included in the article/supplementary material, further inquiries can be directed to the corresponding author.
